# Bioinformatics Analysis of Potential Key Genes in Trastuzumab-Resistant Gastric Cancer

**DOI:** 10.1155/2019/1372571

**Published:** 2019-12-17

**Authors:** Guangda Yang, Liumeng Jian, Xiangan Lin, Aiyu Zhu, Guohua Wen

**Affiliations:** ^1^Department of Cancer Chemotherapy, Zengcheng District People's Hospital of Guangzhou (BoJi-Affiliated Hospital of Sun Yat-sen University), Guangzhou, China; ^2^Department of Neurology, Zengcheng District People's Hospital of GuangZhou (BoJi-Affiliated Hospital of Sun Yat-sen University), Guangzhou, China; ^3^Department of Cancer Chemotherapy, Sun Yat-sen Memorial Hospital of Sun Yat-sen University, Guangzhou, China

## Abstract

**Background:**

This study was performed to identify genes related to acquired trastuzumab resistance in gastric cancer (GC) and to analyze their prognostic value.

**Methods:**

The gene expression profile GSE77346 was downloaded from the Gene Expression Omnibus (GEO) database. Differentially expressed genes (DEGs) were obtained by using GEO2R. Functional and pathway enrichment was analyzed by using Gene Ontology (GO) and Kyoto Encyclopedia of Genes and Genomes (KEGG). Search Tool for the Retrieval of Interacting Genes (STRING), Cytoscape, and MCODE were then used to construct the protein-protein interaction (PPI) network and identify hub genes. Finally, the relationship between hub genes and overall survival (OS) was analyzed by using the online Kaplan-Meier plotter tool.

**Results:**

A total of 327 DEGs were screened and were mainly enriched in terms related to pathways in cancer, signaling pathways regulating stem cell pluripotency, HTLV-I infection, and ECM-receptor interactions. A PPI network was constructed, and 18 hub genes (including one upregulated gene and seventeen downregulated genes) were identified based on the degrees and MCODE scores of the PPI network. Finally, the expression of four hub genes (ERBB2, VIM, EGR1, and PSMB8) was found to be related to the prognosis of HER2-positive (HER2+) gastric cancer. However, the prognostic value of the other hub genes was controversial; interestingly, most of these genes were interferon- (IFN-) stimulated genes (ISGs).

**Conclusions:**

Overall, we propose that the four hub genes may be potential targets in trastuzumab-resistant gastric cancer and that ISGs may play a key role in promoting trastuzumab resistance in GC.

## 1. Introduction

Gastric cancer is the fifth most commonly diagnosed cancer and the third leading cause of cancer-related deaths [1]. The majority of gastric cancer cases are associated with lifestyle factors [2] and infectious agents, including the bacterium Helicobacter pylori [2, 3] and Epstein-Barr virus (EBV) [4, 5]. Although many biomarkers (including HER2, E-cadherin, fibroblast growth factor receptor, PD-L1, and TP53) have been studied as prognostic markers, the 5-year survival rate of gastric cancer remains low [6].

The human epidermal growth factor receptor-2 (HER-2) gene, a proto-oncogene mapped to chromosome 17 (17q12–q21), is frequently found to be amplified and/or overexpressed in gastric cancer [7]. Additionally, HER2 positivity is often associated with a worse prognosis [8, 9]. A phase III trial (the ToGA trial) confirmed that trastuzumab, a HER-2 monoclonal antibody, markedly improved the outcome of HER-2-positive (HER2+) gastric cancer patients [10]. However, a large proportion of patients developed resistance to trastuzumab after continuous treatment despite the effectiveness of this therapeutic [11]. Thus, there is an urgent need to explore the molecular mechanisms of trastuzumab resistance in gastric cancer and to identify effective biomarkers.

Bioinformatics analysis has been widely used to identify key genes in cancer. Interestingly, Piro et al. obtained the gene expression profiles of trastuzumab-sensitive and trastuzumab-resistant cell lines and found that fibroblast growth factor receptor 3 (FGFR3) was associated with trastuzumab resistance in gastric cancer [12]. In the present study, we aimed to further screen DEGs and predict their underlying function by utilizing the same data. More importantly, hub genes affecting trastuzumab resistance in GC patients were identified by a using protein-protein interaction (PPI) network, PPI network modules, and survival analyses.

## 2. Materials and Methods

### 2.1. Microarray Data

The microarray data for GSE77346 deposited by Piro et al. into the GEO database were obtained on the GPL10558 platform (Illumina HumanHT-12 v4.0 Expression BeadChip). The expression profiles are provided for five samples, including one sample of a trastuzumab-sensitive cell line (NCI-N87) and four samples of trastuzumab-resistant cell lines (N87-TR1, N87-TR2, N87-TR3, and N87-TR4).

### 2.2. Identification of DEGs

The web tool GEO2R (https://www.ncbi.nlm.nih.gov/geo/geo2r/) was utilized to screen differentially expressed genes (DEGs) between trastuzumab-resistant and trastuzumab-sensitive gastric cancer cells. These DEGs were identified as important genes that may play an important role in the development of gastric cancer. The cutoff criterion were ∣log fold change (FC)∣ > 3.0 and *P* < 0.01.

### 2.3. Functional and Pathway Enrichment Analysis

We performed Gene Ontology (GO) and Kyoto Encyclopedia of Genes and Genomes (KEGG) pathway enrichment analyses by using the Database for Annotation, Visualization, and Integrated Discovery (DAVID), which is a comprehensive set of functional annotation tools. A *P* value of <0.05 was set as the cutoff criterion.

### 2.4. PPI Network and Module Selection

The Search Tool for the Retrieval of Interacting Genes (STRING) database was used to analyze the PPI network of DEGs. Then, the results were visualized by Cytoscape software. The cutoff criterion for the combined score was >0.4. Subsequently, the degrees of genes in the PPI network and the MCODE plugin in Cytoscape were used to identify hub genes. Genes with degree ≥ 13 or MCODE score ≥ 10 were identified as hub genes. Furthermore, MCODE was used to screen modules in the PPI network.

### 2.5. mRNA Expression of the Hub Genes

UALCAN (http://ualcan.path.uab.edu/index.html) [13], an interactive web portal, can be used to analyze the relative expression of a query gene across tumor and normal samples. The expression levels of the 18 hub genes were analyzed in gastric cancer and normal samples. The calculated *P* value is shown.

### 2.6. Survival Analysis Based on the Hub Genes

The Kaplan-Meier plotter (KM plotter) tool (http://kmplot.com/analysis/index.php?p=background) was used to predict the prognostic value of the hub genes in gastric cancer patients [14]. The patients were divided into two groups according to the particular gene expression level (high vs. low expression). The OS of the two patient groups was then analyzed based on these categories. The hazard ratios (HRs) with 95% confidence intervals and log rank *P* values are shown.

## 3. Results

### 3.1. Identification of DEGs

After data preprocessing, a total of 327 genes were identified, including 128 upregulated genes and 199 downregulated genes.

### 3.2. GO and KEGG Pathway Enrichment Analyses

We conducted GO and KEGG pathway enrichment analyses by using DAVID software. The top five GO terms of the DEGs are shown in Table 1. Regarding biological processes, DEGs were significantly involved in the type I interferon signaling pathway, negative regulation of viral genome replication, cell proliferation, positive regulation of the apoptotic process, and extracellular matrix organization. Regarding the cellular component, DEGs were enriched in the extracellular exosome, intermediate filament, cytoplasm, proteinaceous extracellular matrix, and basolateral plasma membrane. For molecular function, DEGs were enriched in structural molecule activity, calcium ion binding, protein homodimerization activity, 2′-5′-oligoadenylate synthetase activity, and fibronectin binding. The most enriched KEGG pathways included pathways in cancer, signaling pathways regulating stem cell pluripotency, HTLV-I infection, ECM-receptor interaction, and central carbon metabolism in cancer.

### 3.3. Construction of the PPI Network and Module Identification

**Figure 1:**
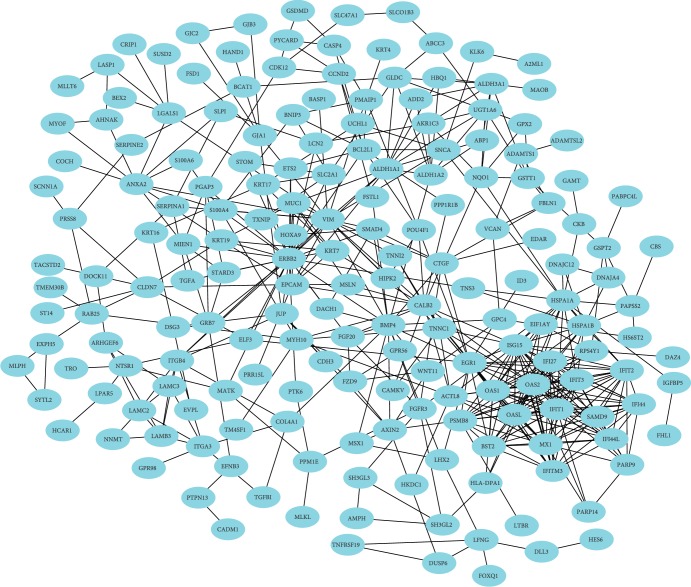
PPI network of the DEGs. Abbreviation: DEGs: differentially expressed genes.

**Figure 2:**
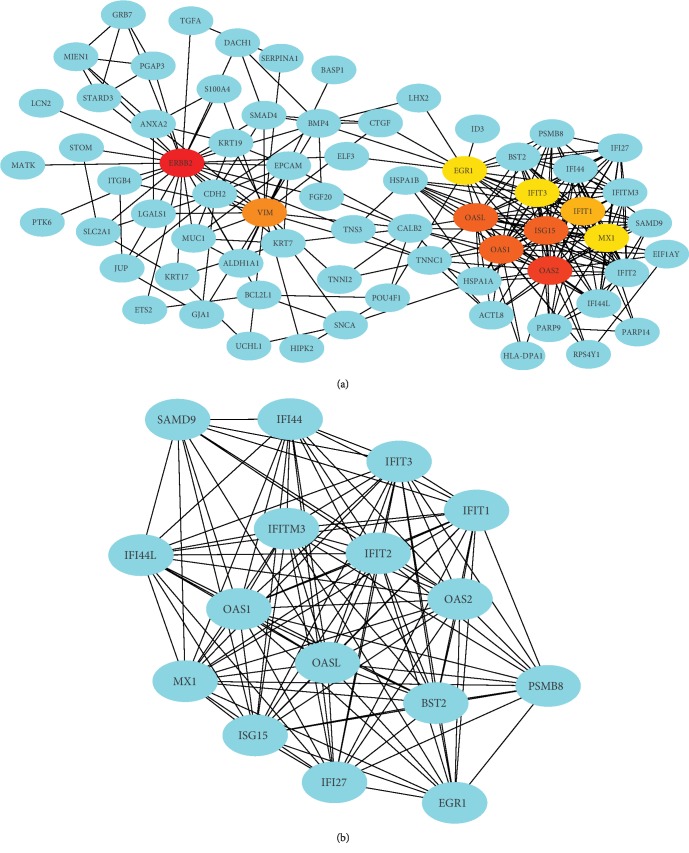
The genes identified by degree (a) and MCODE score (b) in the PPI network.

The PPI network of the DEGs was constructed with 186 nodes and 480 edges by using the STRING database (Figure 1). Degrees ≥ 13 was set as the cutoff criterion. The top 16 genes were ERBB2, OAS2, OASL, ISG15, OAS1, VIM, IFIT1, IFIT2, IFIT3, MX1, EGR1, IFI27, IFI44L, IFITM3, IFI44, and BST2 (Figure 2(a) and Table 2). Subsequently, a significant module with an MCODE score ≥ 10 was selected; this module had 16 nodes and 111 edges, and the included genes were PSMB8, OAS2, OASL, ISG15, OAS1, IFIT1, IFIT2, IFIT3, MX1, EGR1, IFI27, IFI44L, IFITM3, IFI44, BST2, and SAMD9 (Figure 2(b) and Table 2). Thus, 18 genes were identified as hub genes.

### 3.4. mRNA Expression and Survival Analysis

UALCAN database was used to analyze the expression levels of the 18 hub genes. Compared with normal samples, primary gastric cancer samples had higher expression of ERBB2, PSMB8, IFI44, IFI44L, IFIT2, IFIT3, ISG15, OAS1, BST2, IFIT1, IFITM3, MX1, and OAS2. The expression of EGR1 was lower in primary gastric cancer samples than in normal samples. However, no significant difference in VIM, OASL, SAMD9, or IFI27 expression was observed between primary gastric cancer samples and normal samples ([Supplementary-material supplementary-material-1]).

**Figure 3:**
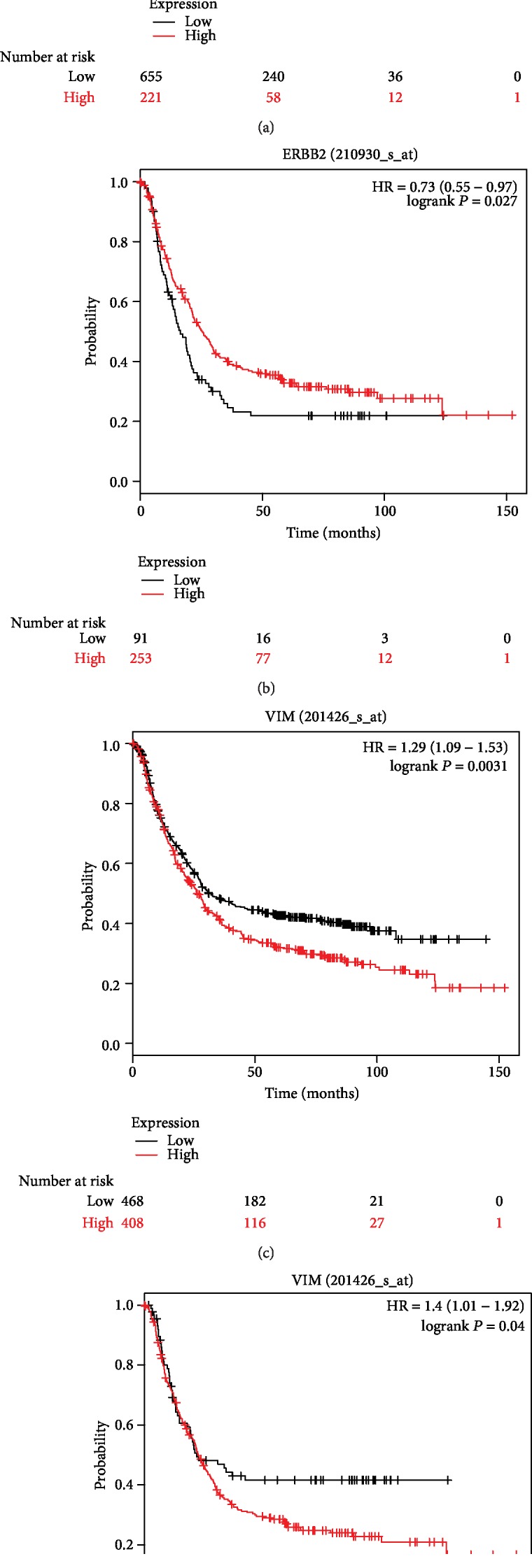
Kaplan-Meier curves depicting the overall survival of all patients with gastric cancer (GC) (a) or HER2-positive (HER2+) gastric cancer (b) with high or low expression of ERBB2 or VIM ((c) GC, (d) HER2+ GC).

**Figure 4:**
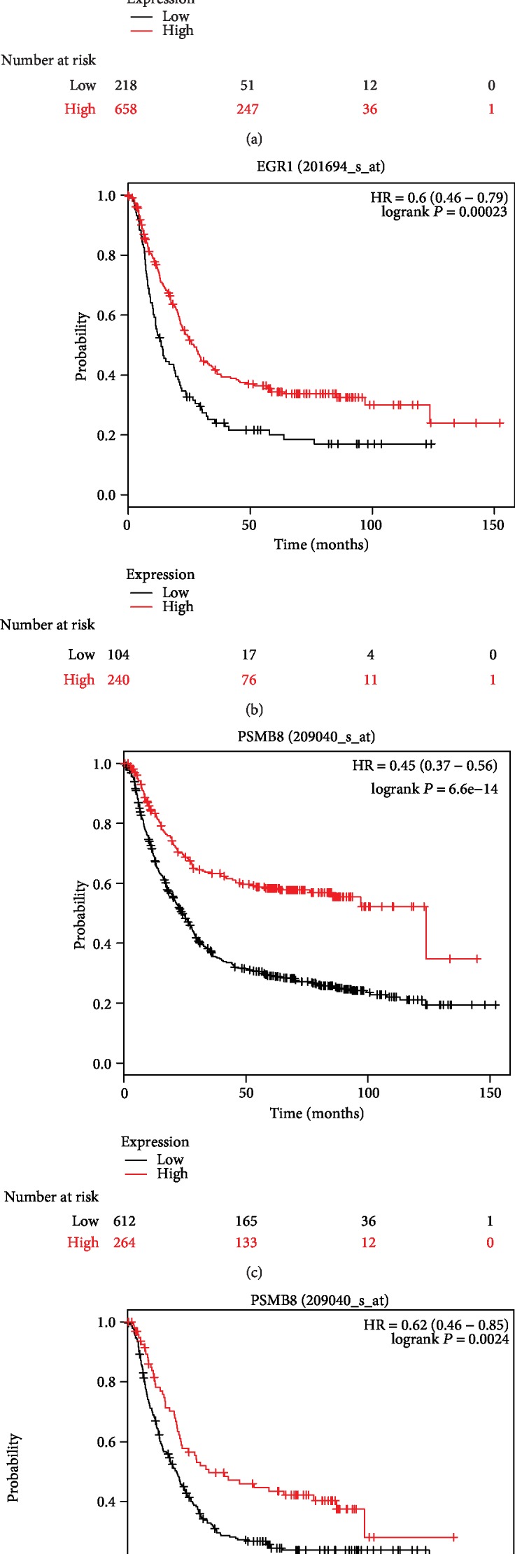
Kaplan-Meier curves depicting the overall survival of all patients with gastric cancer (GC) (a) or HER2-positive (HER2+) gastric cancer (b) with high or low expression of EGR1 or PSMB8 ((c) GC, (d) HER2+ GC).

Kaplan-Meier plotter was used to predict the prognostic value of the 18 hub genes. For all gastric cancer cases, our results showed that high ERBB2 expression was associated with the worse overall survival of GC patients, VIM, IFI44, IFIT2, and MX1 showed similar associations (*P* < 0.05) (Figures 3(a) and 3(c) and Table 3). Additionally, low EGR1 expression was associated with the poorer overall survival of GC patients, and similar associations were found for PSMB8, ASMD9, BST2, IFI27, and IFIT1 (*P* < 0.05) (Figures 4(a) and 4(c) and Table 3). For HER2-gastric cancer, the high expression of ERBB2, VIM, or IFI44 was associated with worse overall survival (*P* < 0.05) (Table 3). In addition, the low expression of PSMB8, IFI44L, IFIT3, ISG15, OAS1, SAMD9, BST2, IFI27, IFIT1, or OAS2 was associated with the poorer overall survival of HER2-GC patients (*P* < 0.05) (Table 3). However, the expression of EGR1, IFIT2, OASL, IFITM3, and MX1 was not associated with the overall survival of HER2-GC patients. For HER2+ gastric cancer, the high expression of VIM, IFI44, IFI44L, IFIT2, IFIT3, ISG15, OAS1, or OASL was associated with worse overall survival (*P* < 0.05) (Figure 3(d) and Table 3). In addition, the low expression of ERBB2, EGR1, or PSMB8 was associated with the poorer overall survival of HER2+ GC patients (*P* < 0.05) (Figures 3(b), 4(b), and 4(d) and Table 3). However, the expression of ASMD9, BST2, IFI27, IFIT1, IFITM3, MX1, and OAS2 was not associated with the overall survival of HER2+ GC patients (Table 3).

We also analyzed the prognostic value of the expression of the 18 hub genes in gastric cancer with different clinicopathological characteristics, including gender, stage, differentiation, and treatment. As shown in [Supplementary-material supplementary-material-1], not all the hub genes had the prognostic value in gastric cancer with different parameters, but some had prognostic value in gastric cancer with specific clinicopathological characteristics. For example, the expression of VIM had prognostic value in almost all categories of gastric cancer. The expression of PSMB8, IFIT2, and IFIT1 had prognostic value in gastric cancer at different stages.

## 4. Discussion

In this study, a total of 327 DEGs were screened, including 128 upregulated genes and 199 downregulated genes. Eighteen genes were identified as hub genes, including one upregulated gene (VIM) and seventeen downregulated genes (ERBB2, PSMB8, OAS2, OASL, ISG15, OAS1, IFIT1, IFIT2, IFIT3, MX1, EGR1, IFI27, IFI44L, IFITM3, IFI44, BST2, and SAMD9). However, survival analysis based on the expression of these genes indicated that only one overexpressed gene (VIM) and three downregulated genes (ERBB2, EGR1, and PSMB8) were significantly associated with the poorer overall survival of HER2+ GC patients.

The data showed that the overexpression of ERBB2 (or HER2) was associated with the worse overall survival of GC patients. Some studies have confirmed that ERBB2 positivity is correlated with a worse prognosis [8, 9], but others have found no relationship between ERBB2 status and prognosis [15, 16]. Therefore, the relationship between ERBB2 status and the prognosis of GC patients remains controversial. However, ERBB2 was found to be downregulated in trastuzumab-resistant cells in the present study, and low ERBB2 expression was associated with a poorer prognosis of HER2+ GC patients. Interestingly, HER2 loss was observed in GC patients treated with trastuzumab in the clinic [17]. This phenomenon indicates that ERBB2 may play an important role in promoting resistance to trastuzumab. The present study found that the overexpression of VIM (vimentin) was correlated with a poorer prognosis of all GC patients, including HER2-GC patients and HER2+ GC patients. Importantly, high VIM expression was associated with a poorer prognosis of almost all categories of GC patients. VIM expression is required for epithelial-mesenchymal transition (EMT) [18]. Some studies have indicated that VIM overexpression is associated with a poorer prognosis among GC patients [19, 20]; this finding is consistent with our results and indicates that VIM may be involved in the development of trastuzumab resistance. The roles of proteasome subunit beta type-8 (PSMB8) and EGR1 in trastuzumab-resistant gastric cancer are controversial. We found that low expression of PSMB8 and EGR1 was associated with a poorer prognosis in all GC patients, including HER2-GC patients and HER2+ GC patients. However, the expression level of PSMB8 was higher in gastric cancer patients than in control patients. A previous study found that increased PSMB8 expression was associated with a lower survival rate of GC patients [21]. Similarly, increased expression of EGR1 was found to be significantly correlated with the depth of invasion and poorer survival of GC patients [22, 23]. Therefore, the roles of PSMB8 and EGR1 in trastuzumab-resistant gastric cancer need to be further investigated. More importantly, many drugs that target ERBB2, including lapatinib [24], trastuzumab [25], and pertuzumab [26], have been investigated. In addition, the ToGA trial confirmed that trastuzumab markedly improves the outcome of HER2+ gastric cancer patients [10]. Moreover, inhibitors of PSMB8 [27] or VIM [28] have been investigated. Carfilzomib, a novel, irreversible proteasome inhibitor, was shown to have potent activity against preclinical models of multiple myeloma [27]. Phenethyl isothiocyanate has been evaluated in trials studying the prevention and treatment of leukemia, lung cancer, tobacco use disorder, and lymphoproliferative disorders [28]. Together, ERBB2, VIM, and PSMB8 may be effective targets in gastric cancer, but more experimental investigations and clinical trials are needed.

IFI44, IFI44L, IFIT2, IFIT3, ISG15, OAS1, and OASL were identified in the subnetwork module. All of these genes are interferon- (IFN-) stimulated genes (ISGs) [29], which mediate the antiviral action of interferon. The interferon-induced protein 44 (IFI44) and interferon-induced protein 44-like (IFI44L) genes belong to the IFI44 family [30]. A previous study found that overexpression of IFI44L decreased doxorubicin chemoresistance and was associated with the better survival of hepatocellular carcinoma patients [31]. The IFN-induced protein with tetratricopeptide repeats (IFITs) family (including IFIT1, IFIT2, IFIT3/4, and IFIT5) is among hundreds of ISGs [32, 33]. Studies have shown that IFIT2 depletion induces cell migration and is associated with poor prognosis in patients with oral squamous cell carcinoma (OSCC) [34, 35]. Similarly, decreased IFIT2 expression predicted poor therapeutic outcomes of GC patients [36]. However, a previous study found that high IFIT3 expression could enhance the chemotherapeutic resistance of pancreatic ductal adenocarcinoma (PDAC) cells and was independently associated with the poor survival of PDAC patients [37].

Another previous study demonstrated that interferon-stimulated gene 15 (ISG15) downregulation could enhance cisplatin resistance via the DNA damage/repair pathway in A549/DDP cells [38]. However, ISG15 was found to be overexpressed in breast carcinoma, and ISG15 overexpression was associated with an unfavourable prognosis [39]. OAS1 and OASL belong to the 2′,5′-oligoadenylate synthetase (2-5OAS) gene family [40]. Many ISGs, including the OASL gene, have been tested for their antiviral specificity [41]. A previous study showed that OASL gene upregulation is involved in the inhibition of lung cancer cell proliferation and apoptosis [42]. Therefore, we propose that ISGs may play an important role in promoting GC resistance to trastuzumab, but the roles of these genes are controversial and need to be further investigated.

EBV plays an important role in gastric carcinogenesis [4, 5]. EBV latent membrane protein (LMP1) and latent membrane protein 2A (LMP2A) regulate the expression of the hub gene EGR1 [43, 44]. LMP2A has been found to suppress the expression of HER2 via the TWIST/YB-1 axis in EBV-associated gastric carcinoma [45]. Importantly, EBV and HER2 may exhibit crosstalk during human gastric carcinogenesis [46]. Interestingly, LMP1 can establish an antiviral state via the induction of ISGs, including OAS [47]. The EBV microRNA BART16 suppresses type I IFN signaling [48]. Thus, EBV may play a key role in gastric carcinogenesis, including in trastuzumab-acquired resistance, by regulating some of the identified hub genes or ISGs.

## 5. Conclusions

In summary, the current study identified genes related to acquired trastuzumab resistance in gastric cancer and analyzed their prognostic value. We found that four hub genes, including ERBB2, VIM, EGR1, and PSMB8, may participate in the development of chemoresistance to trastuzumab. However, the data in the present study were obtained by bioinformatics analysis, and the findings remain to be confirmed by further investigations. Therefore, more experiments are required to ascertain the clinical value of the identified genes as biomarkers and the underlying mechanism.

## Figures and Tables

**Table 1:** Functional and pathway enrichment analyses of the DEGs in trastuzumab-resistant gastric cancer.

	Category	Description	Count	*P* value
GO function	BP	Type I interferon signaling pathway	13	1.04*E* − 09
	BP	Negative regulation of viral genome replication	10	2.46*E* − 08
	BP	Cell proliferation	23	5.99*E* − 07
	BP	Positive regulation of apoptotic process	18	2.51*E* − 05
	BP	Extracellular matrix organization	13	1.92*E* − 04
	CC	Extracellular exosome	90	1.28*E* − 09
	CC	Intermediate filament	11	2.26*E* − 05
	CC	Cytoplasm	122	3.98*E* − 05
	CC	Proteinaceous extracellular matrix	16	5.65*E* − 05
	CC	Basolateral plasma membrane	13	5.98*E* − 05
	MF	Structural molecule activity	17	8.93*E* − 06
	MF	Calcium ion binding	27	4.52*E* − 04
	MF	Protein homodimerization activity	26	1.31*E* − 03
	MF	2′-5′-oligoadenylate synthetase activity	3	1.83*E* − 03
	MF	Fibronectin binding	4	1.06*E* − 02

KEGG pathway
	hsa05200	Pathways in cancer	18	5.47*E* − 04
	hsa04550	Signaling pathways regulating pluripotency of stem cells	8	1.22*E* − 02
	hsa05166	HTLV-I infection	11	1.56*E* − 02
	hsa04512	ECM-receptor interaction	6	1.91*E* − 02
	hsa05230	Central carbon metabolism in cancer	5	2.68*E* − 02

Note: top five terms were selected according to *P* value. Abbreviation: DEGs: differentially expressed genes; GO: gene ontology; BP: biological process; CC: cellular component; MF: molecular function; HTLV-I: human T lymphocyte virus-I; ECM: extracellular matrix.

**Table 2:** The hub genes in the PPI network.

Gene	Regulation	Degree	MCODE scores
ERBB2	Down	29	—
OAS2	Down	24	11.54
OASL	Down	23	11.54
ISG15	Down	23	11.54
OAS1	Down	23	11.54
VIM	Up	21	—
IFIT1	Down	19	11.54
IFIT2	Down	18	11.54
IFIT3	Down	18	11.54
MX1	Down	18	11.54
EGR1	Down	18	12.00
IFI27	Down	14	11.54
IFI44L	Down	14	12.00
IFITM3	Down	14	11.54
IFI44	Down	14	12.00
BST2	Down	14	11.54
PSMB8	Down	—	12.00
SAMD9	Down	—	10.00

**Table 3:** Overall survival analysis based on the expression of the hub genes.

Gene name	ID	OS (all)		OS (HER2-)		OS (HER2+)	
		HR (95% CI)	*P* value	HR (95% CI)	*P* value	HR (95% CI)	*P* value
ERBB2	210930_s_at	1.37 (1.14-1.65)	0.00088	1.33 (1.06-1.66)	0.013	0.73 (0.55-0.97)	0.027
VIM	201426_s_at	1.29 (1.09-1.53)	0.0031	1.52 (1.21-1.9)	0.00027	1.4 (1.01-1.92)	0.04
EGR1	201694_s_at	0.63 (0.52-0.76)	8.5*E* − 07	0.78 (0.61-1)	0.052	0.6 (0.46-0.79)	0.00023
PSMB8	209040_s_at	0.45 (0.37-0.56)	6.6*E* − 14	0.39 (0.3-0.5)	1.7*E* − 13	0.62 (0.46-0.85)	0.0024
IFI44	214059_at	1.46 (1.22-1.74)	0.000028	1.39 (1.1-1.75)	0.0056	1.67 (1.28-2.19)	0.00015
IFI44L	204439_at	0.85 (0.71-1.03)	0.1	0.75 (0.58-0.98)	0.037	1.59 (1.16-2.19)	0.0037
IFIT2	217502_at	1.26 (1.04-1.53)	0.018	0.84 (0.67-1.05)	0.12	1.7 (1.24-2.33)	0.00075
IFIT3	204747_at	1.14 (0.95-1.36)	0.16	0.75 (0.59-0.95)	0.017	1.54 (1.15-2.07)	0.0037
ISG15	205483_s_at	0.86 (0.71-1.03)	0.094	0.73 (0.56-0.93)	0.012	1.5 (1.1-2.05)	0.01
OAS1	202869_at	1.15 (0.97-1.37)	0.1	0.79 (0.63-0.99)	0.039	1.39 (1.07-1.8)	0.013
OASL	210797_s_at	1.16 (0.98-1.37)	0.09	0.84 (0.67-1.05)	0.12	1.41 (1.04-1.92)	0.028
SAMD9	219691_at	0.73 (0.61-0.88)	0.00065	0.57 (0.44-0.74)	0.00002	1.23 (0.94-1.6)	0.14
BST2	201641_at	0.7 (0.58-0.85)	0.00027	0.55 (0.42-0.7)	1.9*E* − 06	1.28 (0.94-1.73)	0.12
IFI27	202411_at	0.7 (0.59-0.84)	0.000096	0.58 (0.46-0.73)	1.8*E* − 06	0.85 (0.63-1.13)	0.26
IFIT1	203153_at	0.73 (0.61-0.87)	0.00061	0.63 (0.49-0.81)	0.00025	1.23 (0.95-1.6)	0.12
IFITM3	212203x_at	1.09 (0.91-1.29)	0.36	0.89 (0.71-1.12)	0.32	1.27 (0.96-1.69)	0.096
MX1	202086_at	1.27 (1.05-1.54)	0.015	1.15 (0.9-1.47)	0.26	1.32 (0.99-1.76)	0.056
OAS2	204972_at	0.91 (0.77-1.08)	0.3	0.77 (0.61-0.96)	0.021	1.25 (0.93-1.67)	0.13

Abbreviation: OS: overall survival; HER2-: human epidermal growth factor receptor-2 negative; HER2+: human epidermal growth factor receptor-2 positive.

## Data Availability

The data used to support the findings of this study are included within the article and the supplementary information files.

## Supplementary Materials

Supplementary Materials Figure S1: the expression levels of ERBB2 (a), VIM (b), EGR1 (c), PSMB8 (d), IFI44 (e), IFI44L (f), IFIT2 (g), IFIT3 (h), ISG15 (i), OAS1 (j), OASL (k), SAMD9 (l), BST2 (m), IFI27 (n), IFIT1 (o), IFITM3 (p), MX1 (q), and OAS2 (r) in gastric cancer (UALCAN database). Red represents primary gastric cancer and blue represents normal samples. Table S1: survival analyses of the hub genes in gastric cancer with different parameters.
