# Corrigendum to “Bioinformatics Analysis of Potential Key Genes in Trastuzumab-Resistant Gastric Cancer”

**DOI:** 10.1155/2020/3147825

**Published:** 2020-12-24

**Authors:** Guangda Yang, Liumeng Jian, Xiangan Lin, Aiyu Zhu, Guohua Wen

**Affiliations:** ^1^Department of Cancer Chemotherapy, Zengcheng District People's Hospital of Guangzhou (BoJi-Affiliated Hospital of Sun Yat-sen University), Guangzhou, China; ^2^Department of Neurology, Zengcheng District People's Hospital of GuangZhou (BoJi-Affiliated Hospital of Sun Yat-sen University), Guangzhou, China; ^3^Department of Cancer Chemotherapy, Sun Yat-sen Memorial Hospital of Sun Yat-sen University, Guangzhou, China

In the article titled “Bioinformatics Analysis of Potential Key Genes in Trastuzumab-Resistant Gastric Cancer” [1], a very similar study published in August 2018 was not cited and discussed [2]. The authors said they used the online tool GEO2R to analyze the GSE77346 dataset in July 2018 and did not conduct another search of the literature after this date. The results differ due to different standards and thresholds.

Firstly, the greatest difference is the different thresholds for obtaining differentially expressed genes (DEGs), which will affect the amount of DEGs obtained and the subsequent data analysis. The authors used ∣ log fold change (FC)∣>3 as the cutoff criterion and obtained 327 DEGs, whereas the previous authors used ∣ log fold change (FC)∣>2 and 849 DEGs were identified.

Secondly, the cutoff criteria of the PPI network and module selection are different, due to the difference of the hub genes.

Thirdly, the authors analyzed the expression levels of the hub genes by using the online UALCAN database (http://ualcan.path.uab.edu/index.html), which is designed to provide easy access to publicly available cancer OMICS data (TCGA and MET500).

Fourthly, the Kaplan-Meier plotter (KM plotter) tool (http://kmplot.com/analysis/index.php?p=background) was used to predict the prognostic value of the hub genes in gastric cancer patients. The authors analyzed the prognostic value of the hub genes in all gastric cancer patients, HER2-negative (HER2-) gastric cancer patients and HER2-positive (HER2+) gastric cancer patients. The genes that have an association with overall survival in HER2+ gastric cancer patients may be potential targets in trastuzumab-resistant gastric cancer.

Fifthly, the authors found that some hub genes were interferon- (IFN-) stimulated genes (ISGs), which may play a key role in promoting trastuzumab-resistance in gastric cancer. The authors discussed the function of the ISGs in detail.
