# Supplementary material for: Bioinformatics Analysis of Potential Key Genes in Trastuzumab-Resistant Gastric Cancer
Source: Dis Markers. 2019 Dec 17;2019:1372571. doi: 10.1155/2019/1372571 (PMC6948351; doi:10.1155/2019/1372571)
Supplement: Supplementary Materials — Figure S1: the expression levels of ERBB2 (a), VIM (b), EGR1 (c), PSMB8 (d), IFI44 (e), IFI44L (f), IFIT2 (g), IFIT3 (h), ISG15 (i), OAS1 (j), OASL (k), SAMD9 (l), BST2 (m), IFI27 (n), IFIT1 (o), IFITM3 (p), MX1 (q), and OAS2 (r) in gastric cancer (UALCAN database). Red represents primary gastric cancer and blue represents normal samples. Table S1: survival analyses of the hub genes in gastric cancer with different parameters. [file 1372571.f1.pdf]

Supplementary Materials:

Figure S1. The expression levels of ERBB2(a), VIM (b), EGR1(c), PSMB8 (d), IFI44 (e), IFI44L(f), IFIT2 (g), IFIT3 (h), ISG15 (i), OAS1 (j), OASL (k), SAMD9 (l), BST2 (m), IFI27 (n), IFIT1 (o), IFITM3 (p), MX1(q) and OAS2 (r) in gastric cancer (UALCAN data base). Red represents primary gastric cancer, Blue represents normal samples.

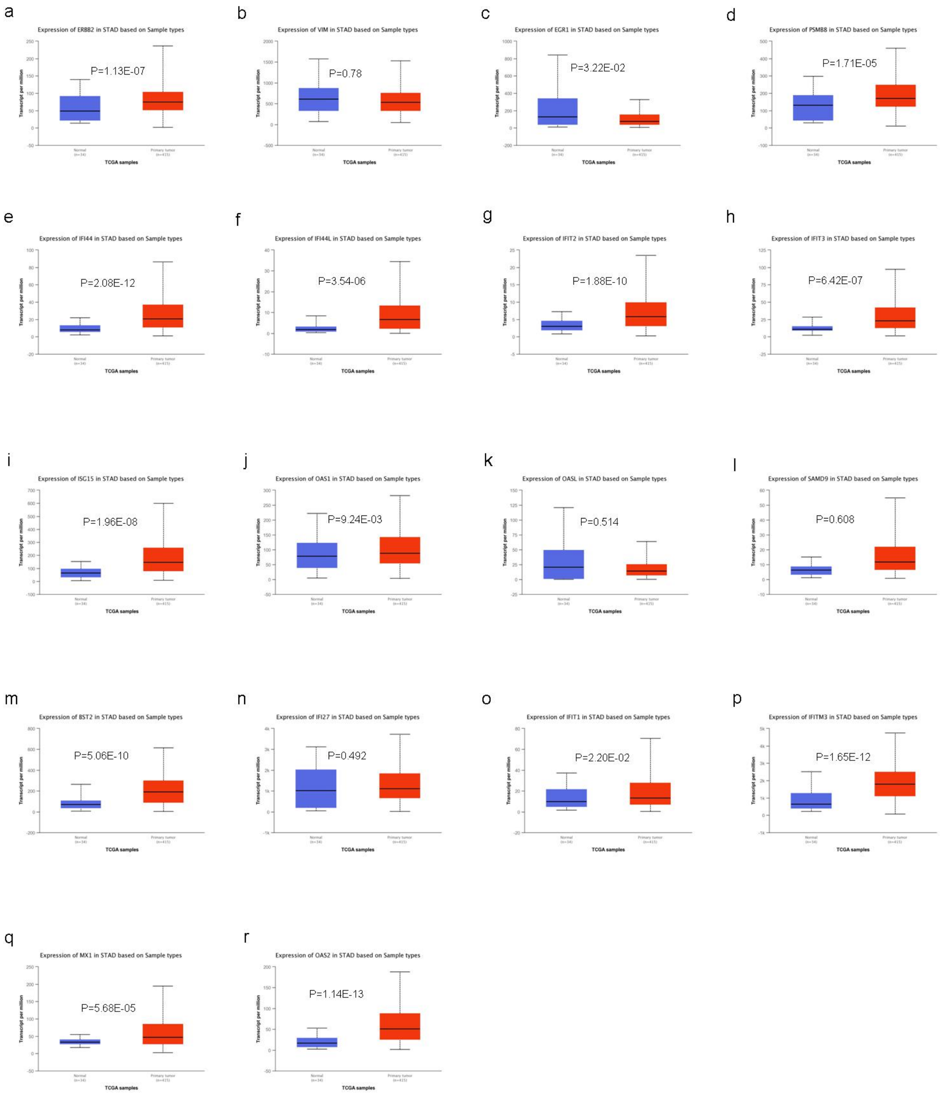

TABLE S1. Survival analyses of the hub genes in gastric cancer with different parameters.

TABLE S1. Survival analyses of the hub genes in gastric cancer with different parameters.

Parameters	ERBB2 (210930_at)		VIM (201426s-at)		EGR1 (201694_s_at)		PSMB8 (209040_s_at)		IFI44 (214059_at)		IFI44L(204439_at)	
	HR (95% CI)	P-value	HR (95% CI)	P-value	HR (95% CI)	P-value	HR (95% CI)	P-value	HR (95% CI)	P-value	HR (95% CI)	P-value
**Gender**												
female	1.38(0.96-1.98)	0.084	1.61(1.13-2.3)	0.0084	0.66(0.45-0.97)	0.032	0.44(0.29-0.67)	7.20E-05	1.81(1.26-2.6)	0.0011	1.29(0.91-1.84)	0.15
male	1.49(1.19-1.86)	5.00E-04	1.35(1.09-1.68)	0.0051	0.53(0.42-0.67)	2.70E-08	0.47(0.37-0.6)	1.10E-10	1.41(1.13-1.74)	0.0018	0.81(0.64-1.02)	0.073
**Stage**												
I	3.99(1.44-11.04)	0.004	2.04(0.73-5.68)	0.17	2.31(0.79-6.74)	0.11	0(0-Inf)	0.0022	0.42(0.15-1.16)	0.083	0.43(0.16-1.14)	0.081
II	1.59(0.78-3.24)	0.2	2.22(1.19-4.16)	0.01	1.77(0.88-3.53)	0.1	0.34(0.19-0.63)	0.00034	1.51(0.82-2.77)	0.18	2.16(1.06-4.37)	0.029
III	1.65(0.18-2.31)	0.0032	1.51(1.13-2.01)	0.0047	0.79(0.59-1.05)	0.11	0.55(0.41-0.75)	0.00012	1.26(0.93-1.7)	0.13	0.83(0.6-1.13)	0.23
IV	0.8(0.54-1.17)	0.25	1.91(1.28-2.85)	0.0012	1.29(0.88-1.91)	0.19	0.49(0.33-0.73)	0.00039	0.62(0.4-0.97)	0.033	0.61(0.39-0.95)	0.027
**Differentiation**												
poorly differentiated	1.56(1.04-2.33)	0.029	0.61(0.41-0.91)	0.015	1.19(0.77-1.82)	0.43	0.64(0.43-0.95)	0.025	1.5(0.99-2.29)	0.056	0.71(0.45-1.14)	0.15
moderately differentiated	1.84(0.96-3.53)	0.061	2.01(1.01-4.03)	0.044	1.34(0.63-2.84)	0.45	0.72(0.36-1.41)	0.33	0.74(0.39-1.42)	0.36	1.35(0.63-2.87)	0.44
well differentiated	2.18(0.84-5.7)	0.1	2.74(1.12-6.68)	0.021	2.76(0.93-8.23)	0.057	0.46(0.19-1.15)	0.091	3.15(1.31-7.55)	0.0069	3.82(1.38-10.6)	0.0059
**Treatment**												
surgery alone	1.4(1.03-1.89)	0.03	1.45(1.08-1.93)	0.011	1.37(1.03-1.83)	0.03	0.44(0.31-0.63)	3.50E-06	1.34(0.96-1.89)	0.089	0.76(0.54-1.07)	0.12
5-FU based adjuvant	0.67(0.47-0.95)	0.022	0.62(0.44-0.89)	0.0077	0.46(0.3-0.7)	0.00025	1.35(0.91-2)	0.13	2.26(1.5-3.42)	7.00E-05	0.89(0.62-1.29)	0.55
other adjuvant	0.51(0.17-1.54)	0.22	4.77(1.59-14.31)	0.0021	1.95(0.57-6.68)	0.28	0.09(0.01-0.66)	0.0027	4.75(1.1-20.46)	0.021	0.29(0.07-1.25)	0.078
**Parameters**	**IFIT2 (217502_at)**		**IFIT3 (204747_at)**		**ISG15 (205483_s_at)**		**OAS1 (202869_at)**		**OASL (210797_s_at)**		**ASMD9 (219691_at)**	
	HR (95% CI)	P-value	HR (95% CI)	P-value	HR (95% CI)	P-value	HR (95% CI)	P-value	HR (95% CI)	P-value	HR (95% CI)	P-value
**Gender**												
female	1.61(1.05-2.48)	0.029	1.27(0.88-1.83)	0.21	0.8(0.54-1.17)	0.25	1.25(0.88-1.78)	0.21	1.36(0.96-1.93)	0.082	0.56(0.38-0.82)	0.003
male	1.34(1.08-1.67)	0.0075	1.19(0.96-1.48)	0.1	0.87(0.69-1.11)	0.27	0.86(0.7-1.07)	0.17	1.21(0.94-1.56)	0.14	0.69(0.56-0.86)	0.00092
**Stage**												
I	0.32(0.12-0.89)	0.022	0.41(0.14-1.18)	0.087	0.33(0.11-1.03)	0.046	0.4(0.15-1.09)	0.064	0.37(0.14-0.99)	0.039	0.18(0.04-0.78)	0.0096
II	1.73(0.95-3.16)	0.068	2.04(0.91-4.61)	0.078	0.61(0.28-1.3)	0.19	1.91(1.04-3.5)	0.034	1.94(1.07-3.52)	0.026	0.66(0.34-1.26)	0.2
III	1.38(1.03-1.85)	0.029	1.28(0.96-1.71)	0.097	0.82(0.59-1.15)	0.25	0.7(0.53-0.94)	0.016	0.76(0.55-1.05)	0.092	0.55(0.4-0.74)	0.00009
IV	1.72(1.15-2.56)	0.0072	0.75(0.51-1.11)	0.15	0.73(0.5-1.07)	0.1	0.79(0.51-1.21)	0.28	0.76(0.49-1.17)	0.21	1.22(0.83-1.79)	0.3
**Differentiation**												
poorly differentiated	1.49(0.92-2.42)	0.1	0.73(0.45-1.18)	0.2	1.31(0.85-2.02)	0.23	1.61(1.05-2.47)	0.026	0.74(0.47-1.17)	0.2	0.72(0.47-1.1)	0.13
moderately differentiated	1.73(0.76-3.95)	0.19	1.51(0.78-2.94)	0.22	2.65(1.23-5.71)	0.0096	0.62(0.29-1.32)	0.21	1.52(0.73-3.15)	0.26	2.01(1.05-3.87)	0.033
well differentiated	10.5(.4-78.81)	0.0046	4.74(1.38-16.24)	0.0065	2.35(0.79-7)	0.11	0.51(0.2-1.32)	0.16	0.49(0.19-1.27)	0.13	1.64(0.55-4.88)	0.37
**Treatment**												
surgery alone	0.9(0.66-1.23)	0.52	0.77(0.57-1.05)	0.1	0.65(0.46-0.94)	0.02	0.78(0.59-1.04)	0.095	0.79(0.59-1.05)	0.11	0.64(0.46-0.87)	0.0049
5-FU based adjuvant	0.55(0.38-0.79)	0.0011	1.31(0.93-1.85)	0.12	2.0(1.38-2.88)	0.00016	1.69(1.16-2.46)	0.006	0.73(0.51-1.05)	0.089	0.72(0.5-1.06)	0.095
other adjuvant	2.63(0.88-7.88)	0.072	0.59(0.24-1.44)	0.24	0.59(0.24-1.49)	0.26	0.52(0.22-1.25)	0.14	0.36(0.11-1.24)	0.093	0.4(0.15-1.11)	0.07
**Parameters**	**BST2 (201641_at)**		**IFI27 (202411_at)**		**IFIT1 (203153_at)**		**IFITM3 (212203x_at)**		**MX1 (202086_at)**		**OAS2 (204972_at)**	
	HR (95% CI)	P-value	HR (95% CI)	P-value	HR (95% CI)	P-value	HR (95% CI)	P-value	HR (95% CI)	P-value	HR (95% CI)	P-value
**Gender**												
female	0.42(0.26-0.68)	0.00031	0.57(0.39-0.86)	0.0058	0.82(0.56-1.18)	0.28	0.88(0.61-1.25)	0.47	1.3(0.88-1.91)	0.19	0.68(0.48-0.97)	0.032
male	0.79(0.63-1)	0.049	0.75(0.6-0.93)	0.008	0.72(0.58-0.91)	0.0045	1.23(0.98-1.55)	0.069	1.3(1.03-1.65)	0.03	1.1(0.89-1.36)	0.39
**Stage**												
I	0.19(0.04-0.84)	0.014	0.42(0.16-1.13)	0.076	0.36(0.13-0.96)	0.032	0.51(0.19-1.42)	0.19	0.3(0.07-1.31)	0.088	0.27(0.09-0.85)	0.017
II	0.69(0.37-1.25)	0.22	0.62(0.32-1.18)	0.14	2.41(1.07-5.43)	0.028	0.72(0.38-1.36)	0.31	2.06(0.87-4.89)	0.094	1.66(0.9-3.09)	0.1
III	0.74(0.55-0.98)	0.036	0.6(0.45-0.81)	0.00051	0.62(0.45-0.85)	0.0029	1.79(1.26-2.55)	0.001	1.39(1-1.94)	0.049	0.78(0.55-1.1)	0.15
IV	0.67(0.44-1.04)	0.072	0.59(0.4-0.87)	0.0066	0.72(0.48-1.08)	0.11	0.77(0.52-1.13)	0.17	0.43(0.43-1.03)	0.068	0.67(0.46-0.99)	0.042
**Differentiation**												
poorly differentiated	0.55(0.37-0.82)	0.0026	0.71(0.47-1.05)	0.086	0.78(0.49-1.25)	0.31	1.38(0.92-2.06)	0.11	0.82(0.54-1.25)	0.35	0.66(0.43-1.01)	0.055
moderately differentiated	2.26(0.99-5.18)	0.048	1.83(0.95-3.51)	0.068	1.45(0.76-2.77)	0.26	0.6(0.27-1.32)	0.2	2.15(1.07-4.31)	0.027	1.87(0.9-3.89)	0.091
well differentiated	4.11(1.12-14.13)	0.015	0.61(0.26-1.44)	0.25	2.78(1.077.22)	0.028	4.71(1.09-20.3)	0.022	2.53(1.04-6.15)	0.035	0.62(0.26-1.48)	0.28
**Treatment**												
surgery alone	0.57(0.39-0.84)	0.0035	0.57(0.4-0.82)	0.0017	0.86(0.64-1.16)	0.33	1.22(0.89-1.68)	0.21	0.8(0.58-1.09)	0.16	0.74(0.55-0.98)	0.037
5-FU based adjuvant	0.77(0.52-1.14)	0.2	1.55(0.9-2.21)	0.015	0.68(0.48-0.96)	0.026	1.5(1.05-2.16)	0.026	1.68(1.17-2.41)	0.0044	1.72(1.2-2.47)	0.003
other adjuvant	1.84(0.76-4.46)	0.17	0.11(0.01-0.81)	0.0084	1.54(0.56-4.25)	0.4	3.14(0.92-10.72)	0.054	0.53(0.19-1.45)	0.21	0.45(0.16-1.24)	0.11
